# Effect of distance to health facility on the maintenance of INR therapeutic ranges in rheumatic heart disease patients from Cape Town: no evidence for an association

**DOI:** 10.1186/s12913-015-0890-4

**Published:** 2015-06-05

**Authors:** Dylan D Barth, Liesl J Zühlke, Alexia Joachim, Tyler Hoegger, Bongani M Mayosi, Mark E Engel

**Affiliations:** Department of Medicine, Groote Schuur Hospital and University of Cape Town, J46.43 Old Main Building, Private Bag, 7700 Rondebosch, South Africa; Department of Paediatrics, Red Cross War Memorial Children’s Hospital and University of Cape Town, Cape Town, South Africa; Skiza Consulting Ltd., Cape Town, South Africa

**Keywords:** GIS, INR, RHD, Distance decay

## Abstract

**Background:**

Lack of adherence to international normalised ratio (INR) monitoring in rheumatic heart disease (RHD) patients is a contributor to cardio-embolic complications. This population-based observational study investigated whether the distance between home and an INR clinic affects the maintenance of therapeutic INR in RHD patients on warfarin.

**Methods:**

Residential addresses, INR clinics, and INR results of patients with RHD were extracted from the Cape Town component of the Global Rheumatic Heart Disease Registry (REMEDY) database. Addresses of homes and INR clinics were converted to geographical coordinates and verified in ArcGIS 10®. ArcGIS 10® and Google Maps® were used for spatial mapping and obtaining shortest road distances respectively. The travel distance between the home and INR clinic was correlated with time within therapeutic range (TTR) using the Rosendaal linear interpolation method, and with the fraction of INR within range, based on an average of three INR readings of patients and compared with recommended therapeutic ranges.

**Results:**

RHD patients (n = 133) resided between 0.2 km and 50.8 km (median distance, 3.60 km) from one of 33 INR clinics. There was no significant difference in the achievement of the therapeutic INR between patients who travelled a shorter distance compared to those who travelled a longer distance (in range = 3.50 km versus out of range = 3.75 km, *p* = *0.78*). This finding was the same for patients with mechanical valve replacement (n = 105) (3.50 km versus 3.90 km, *p* = *0.81*), and native valves (3.45 km versus 2.75 km, *p = 0.84*).

**Conclusions:**

There is no association between the maintenance of INR within therapeutic range amongst RHD patients in Cape Town and distance from patients’ residence to the INR clinic.

## Background

Various factors for non-adherence to health-related interventions and monitoring have been suggested by the World Health Organisation (WHO), including distance decay [1, 2] Distance decay is a term given to describe the effect of distance on interactions observed between two locations; in this case, it is observed that travel distance to health services is inversely proportional to the rate of service use, thereby resulting in geographical health care disparities [3]. A recent study on distance decay and clinic utilization in South Africa identified the need to improve the quality of care in clinics in poorer areas, thus potentially minimizing the distance travelled to access health care in better resourced areas [4]; an earlier study by Tanser et al. found evidence of distance decay in primary health care utilization in South Africa [5]. Elsewhere, a study on delivery care utilization in Vietnam found an increased risk of neonatal mortality among mothers living farthest away from a health facility [6]. Another study conducted in Kenya documented a correlation between median distance to a health facility with severity of illness; furthermore, the rate of clinic visits decreased linearly at 0.5 km intervals and stabilised after 4 km [7].

Rheumatic heart disease (RHD) is a condition characterised by permanent heart valve damage, which may, in severe cases, require corrective heart valve surgery [8, 9]; however, there is an additional risk of blood clot formation and/or embolism, which may result in stroke [10]. A higher risk of stroke is also associated with another complication, namely atrial fibrillation [11]. Anticoagulant drugs such as warfarin are thus prescribed. Anticoagulation therapy, which interferes with the clotting process is highly effective at reducing the risk of stroke [12, 13] but requires regular laboratory-guided adjustments of the dose, as response to treatment is affected by interactions with food and drugs [14, 15]. Monitoring is done through the international normalized ratio (INR), which represents the comparison of clotting times in patients who have been prescribed anticoagulants against the clotting times in healthy individuals. The safety and effectiveness of anticoagulant therapy is crucially dependent on maintaining the INR within the therapeutic range [16, 17].

To the best of our knowledge, no studies exist on the effect of distance decay on the maintenance of INR therapeutic ranges among RHD patients. The primary aim of this study, therefore, was to evaluate the effect of distance on the maintenance of INR in the therapeutic range amongst RHD patients on anticoagulant therapy. Specifically, this study aimed to investigate whether the distance of a patient’s residence from their INR clinic impacts on maintaining INR levels within the recommended therapeutic ranges over the scheduled monthly visits to the clinic.

## Methods

### Study design

This was a population-based observational sub-study nested within the Groote Schuur Hospital (GSH) cohort of the Global Rheumatic Heart Disease Registry (REMEDY) study, a multicenter, prospective hospital-based RHD registry [18]. The REMEDY project aims to provide comprehensive, contemporary data on patients with RHD so as to aid the development of strategies to prevent and manage RHD and its complications. This study is a retrospective analysis of two methods for determining INR control and evaluating the time within therapeutic range (TTR). The TTR was calculated using the fraction of INRs in range, based on the average of 3 INR readings [19] and the Rosendaal linear interpolation method [20]. This study was approved by the University of Cape Town Human Research Ethics Committee, and all participants gave written informed consent or assent.

### Recruitment and enrolment

All GSH patients diagnosed with RHD and treated with warfarin in the REMEDY study, were eligible to participate in the study. Patients were excluded if their address data were incomplete at their 12-month follow-up visit, if they had not had a follow-up visit with INR data being recorded, or if they resided outside of the Cape Metropole area, which is located in the Western Cape province of South Africa.

### Data

Patient data including demographic details, residential addresses and clinical data, such as diagnosis of RHD, INR readings and referral INR clinics were extracted from REMEDY patient records and captured into a purpose-designed database. Evidence-based clinical guidelines guide INR therapeutic level ranges for patients, which are between 2.00 and 3.00 for patients without valve replacements and between 2.5 and 3.5 for patients with mechanical prosthetic valve replacements [16]. Patient addresses were converted to geographical coordinates obtained from Google Earth®, while government clinics were mapped using information supplied by the Department of Surveying and Mapping in Mowbray, Cape Town.

### Geographical information systems

This study utilized Geographical Information System (GIS) software, ArcGIS 10®, for capturing, manipulating, managing, analysing and displaying spatial and non-spatial data. In brief, GIS overlaps layers of information within a geographical area to give a better understanding from a composite viewpoint [21]. Information about geographical features (roads, boundaries etc.) was accessed from the ESRI database (https://www.arcgis.com). Data were projected in the Universal Transverse Mercator (UTM) system. The shortest road travel distances between homes and the health facility utilized by patients for INR monitoring were calculated using Google Maps (https://maps.google.co.za/maps?hl=en) and entered into the study database. Linear distances between the patient’s home and closest health facility were calculated using the “near” function in the “proximity” toolbox in ArcMap 10®. The geocoded addresses were checked in ArcGIS for errors in coordinate data. Distances mentioned are all one-way distances.

### Statistical analysis

Statistical analyses and data handling were performed using STATA® version 11 and ArcGIS® version 10. We evaluated INR readings as a whole, as well as stratified according to valve type. Pearson’s chi-squared test and Mann–Whitney *U* test were used for group comparisons with a p-value of < 0.05 considered as being significant. The road travel distances between individual residential addresses and INR clinics were compared with the average of the latest three INR measurement readings of the patient. Standard deviation (SD) and 95 % confidence intervals (CI) were used to evaluate data dispersion.

## Results

One-hundred and thirty-three patients enrolled at GSH in the REMEDY database met the inclusion criteria. Eighty-three percent of the cohort was female (n = 110) and 79 % of patients (n = 105) had had mechanical valve replacements (MVRs). Co-morbidities included hypertension (27 %), stroke (25 %) and diabetes (19 %) (Table 1). Among the participants excluded due to unavailability of address details or INR data, 185 (83 %) were female and 81 (36 %) had had MVRs; the median age was 46 years (16–79 years).

**Table 1:** Baseline characteristics of participants

	Number	Percent
Patients who completed >12 month follow-up and for whom address and INR data were available	133	
Average age (*SD*)	53 years (1.25)	
Female	110	*83*
No of patients with mechanical valve replacement	105	*79*
No of patients with non-mechanical valve replacement	28	*21*
*Co-morbidities*	n/N^a^	*%*
Hypertension	27/99	*27*
Stroke	28/111	*25*
Diabetes	19/98	*19*

*N* total number of patients, *%* percentage, *SD* standard deviation, *n* number of patients who suffer from co-mobidities

^a^Data were not available on all patients

**Fig. 1:**
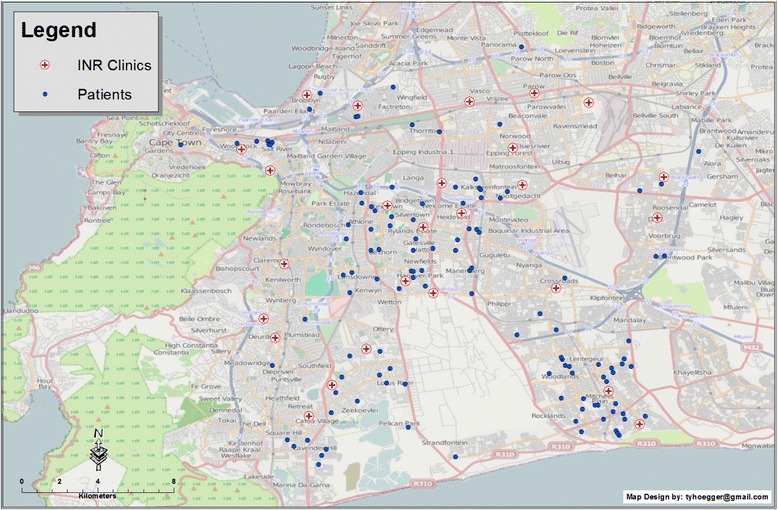
Map of the Cape Metropole showing patients’ residences and the clinics utilized for INR monitoring. Each blue circle represents a patient’s residence; each red cross represents the INR clinics utilized by RHD patients

**Fig. 2:**
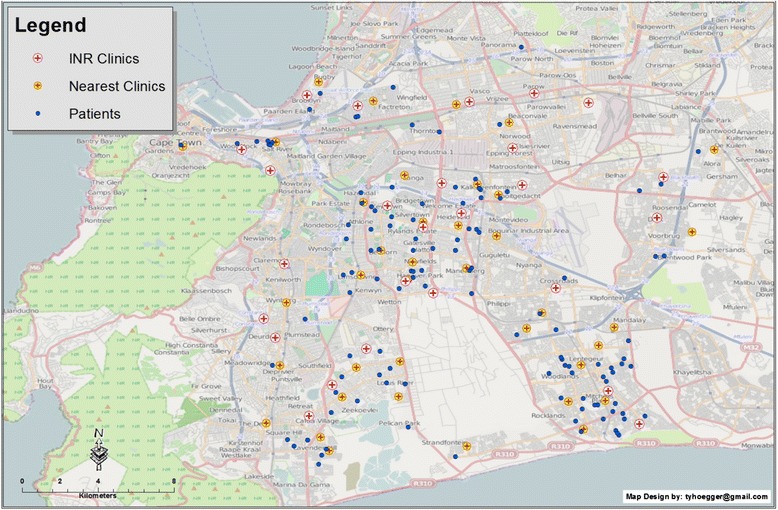
Map of the Cape Metropole showing patients’ residences (blue circles), the clinics utilized for INR monitoring (red cross within white circle) and clinics that could potentially be used for INR monitoring within closer proximity to patients’ residences (red cross within a yellow circle)

Figures 1 and 2 represent the spatial orientation of the RHD patients’ residences and the INR clinics in the Cape Metropole region. INR monitoring was conducted among 33 clinics across the Cape Metropole. Road distances were not normally distributed; the median distance between the patient’s residences and their respective clinics was 3.60 km (range, 0.2 km–50.8 km). Figure 2 displays the clinics utilized by RHD patients for INR monitoring as well as clinics not currently used for INR monitoring. The geographical distribution of these clinics indicates that patients could instead be utilizing clinics closer to their residences.

Three-hundred and thirty-four INR values were included in the analysis. The mean TTR over the study period for the fraction of INR’s in range for MVR and non-MVR patients was 0.42 (95 % confidence interval (CI), 0.31; 0.53) and 0.67 (95 % CI, 0.53; 0.78) respectively. Table 2 provide the details pertaining to seven clinics utilized by 3 or more patients for INR monitoring, indicating the number of patients who maintained therapeutic levels according to whether or not they had had a MVRs. Overall, patients with mechanical valves were less likely to be within their therapeutic INR range than patients with native valves (OR: 0.32, 95 % CI: 0.11, 0.86). Using the Rosendaal method, TTR values for MVR and non-MVR patients were 0.38 and 0.58 respectively.

**Table 2:** Shortest median road distance from patients’ homes to health facilities

	Obs	No. in INR range		Median km (range)
		MVR = no	MVR = yes	
All clinics	133	20/28 (71)	47/105 (45)	3.60 (0.2–50.8)
Individual clinics^a^				
GSH	35	5/7 (71)	15/28 (54)	18.2 (1.6–50.8)
Dr Abdurahman CHC	10	0	3/10 (30)	2.95 (1.0–5.4)
Mitchell’s Plain CHC	21	1/4 (25)	7/17 (41)	2.8 (1.2–5.6)
Heideveld CHC	10	1/2 (50)	3/8 (38)	2.75 (1.6–6.5)
Hanover Park CHC	8	1/1 (100)	4/7 (57)	1.1 (0.2–9.1)
Lady Michaelis CHC	5	1/2 (50)	1/3 (33)	6.4 (1.8–14.0)
Woodstock CHC	4	1/2 (50)	1/2 (50)	1.55 (0.4–14.9)

*Obs* observations, *No* number, *km* kilometres, *MVR* mechanical valve replacement, *GSH* Groote Schuur Hospital, *CHC* community health clinic

^a^Only clinics with 3 or more patients were evaluated

Among all the patients in our cohort, those who maintained their INR within recommended therapeutic levels travelled a shorter median distance to their designated INR clinic (3.50 km versus 3.75 km) (Table 3). However, this association was not statistically significant (*p = 0.78*). This trend was also observed within the MVR subset of patients (n = 105) (in range = 3.50 km versus out of range = 3.90 km, *p = 0.81*). This trend however, contrasted with patients with native valves (n = 28), where the patients who maintained therapeutic levels travelled a further median distance to their designated clinics (3.45 km versus 2.75 km, *p = 0.84*).

**Table 3:** Median shortest road travel distance from patients’ homes to health facilities, overall and by valve type

	n (%)	Median km	
All patients			
Out of therapeutic range	66 (49.6)	3.75 (0.4–50.8)	*p = 0.78*
In therapeutic range	67 (50.4)	3.50 (0.2–30.8)	
Total	133 (100)		
Mechanical valve = yes			
Out of therapeutic range	58 (55)	3.90 (0.4–50.8)	*p = 0.81*
In therapeutic range	47 (45)	3.50 (0.2–30.8)	
Total	105 (100)		
Mechanical valve = no			
Out of therapeutic range	8 (29)	2.75 (1.2–18.2)	*p = 0.84*
In therapeutic range	20 (71)	3.45 (1.0–24.0)	
Total	28 (100)		

*n* number of observations, *%* percentage, *km* kilometres

**Fig. 3:**
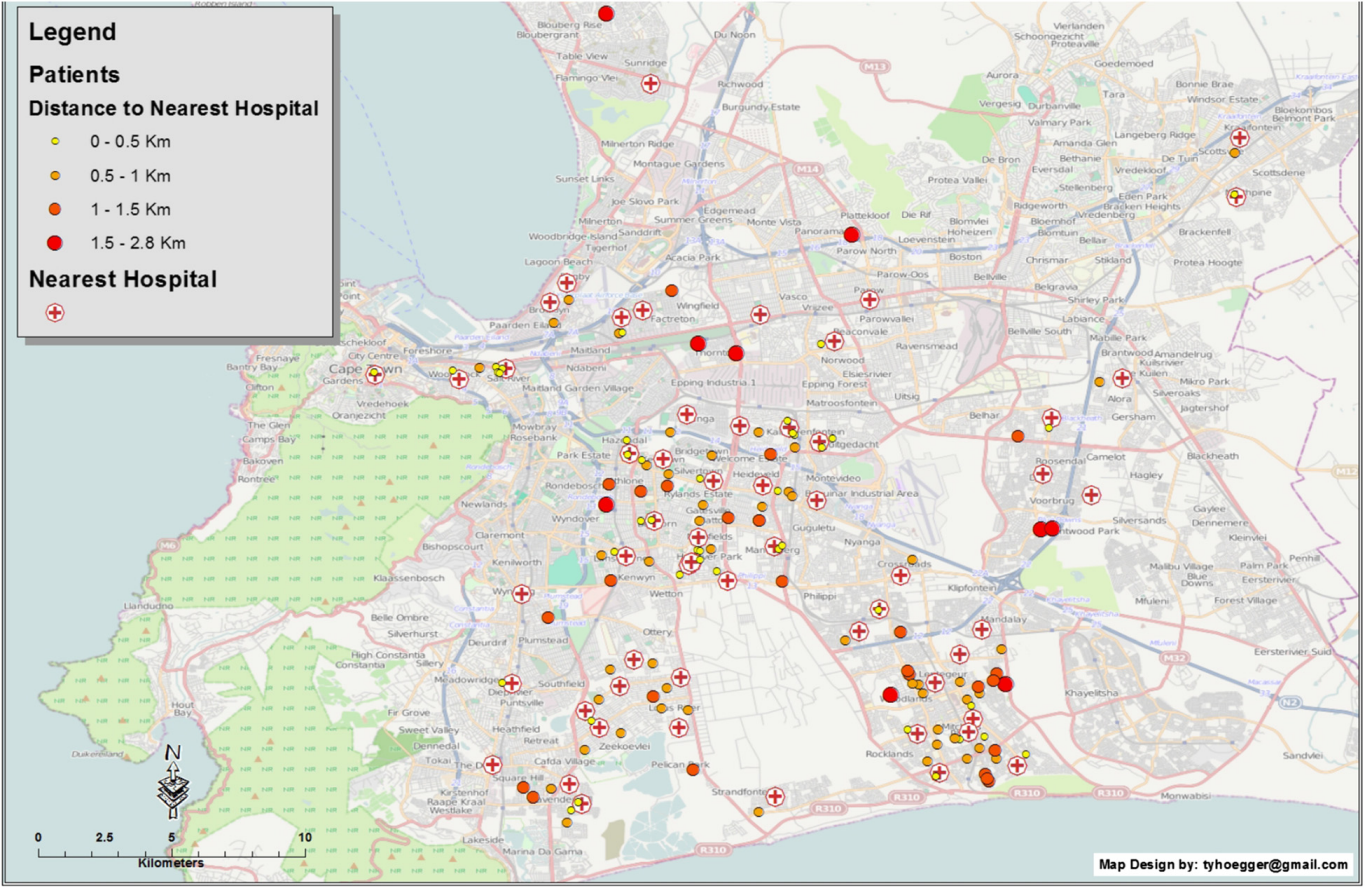
Map showing the patients’ residences (blue circles) and INR clinics (red cross within circle) using graduated symbology to represent the distance to the nearest clinic for potential INR monitoring, represented by graduated circles in size and colour ranging from yellow (0–0.5 km) to red (1.5–2.8 km)

Figure 3 depicts the distance of the closest clinic relative to the patient’s home address; the median distance from the patients’ residence to the closest clinic is 0.67 km (0.08–2.80).

## Discussion

Our study demonstrates that was no difference in the median distance from an RHD patient’s residence to their designated INR clinic in respect of those who maintained their INR readings within the therapeutic range versus those who did not (3.50 km versus 3.75 km; *p = 0.78*). Patients attending Groote Schuur Hospital (GSH), the only tertiary hospital included among the INR clinics, travelled a median distance of 18.2 km (1.6 km–50.8 km), which was noticeably further than the median road distance of 3.60 km travelled by patients overall to treatment facilities. Reasons for this may include a possible perception of higher quality care or convenience associated with GSH, if located near to a place of employment [22]. The overall proportion of patients within the target INR therapeutic range was 50.3 %. Given that target INR differs according to whether patients had undergone a mechanical valve replacement (MVR) or not (i.e., 2.0–3.0 for no or biological valve replacement, and 2.5–3.5 for MVR), we also considered MVR patients separately. No statistically significant association was established between distance and maintenance of INR therapeutic levels among MVR patients. The TTR values obtained using the Rosendaal interpolated method confirmed the trends calculated by the fraction method. However, it must be borne in mind that the Rosendaal method tends to deflate the TTR, for reasons which are unclear [19].

Participants for our study needed to have at least two INR readings, to reside within the geographical metropolitan area served by the hospital, and to have complete address information; this may represent selection bias. Participants excluded from the study were similar as regards gender and mean age; of these, 79 % had MVRs. Unfortunately, we did not have correlates for missing data, such as socio-economic status, and thus could not perform correlation analysis to impute the likelihood of distance barriers.

We made use of both linear and road distances in our analysis. The shortest road travel distances between patients’ homes and health facilities were measured using Google Maps®. The median distance to the closest health facility from patients’ residences, using linear distance measurement was 0.67 km. The extent to which the straight-line distance measurement is used as a proxy for true distance is sometimes questionable and raises concern, when a particular geographical area of study has diverse features, for example, in vast mountainous areas, where there is limited road access. In this study, the geographical area has a good road infrastructure and is not affected by diverse geographical features, thus we are confident that the straight-line distance measurement used in this setting is closely representative of the true road distance travelled. Furthermore, straight-line distances have previously been used in many studies including those from Vietnam and Kenya discussed below.

Published studies that evaluated the effect of patients’ travel distance on utilization of health services report results, which differ from ours. A neonatal mortality study conducted in Vietnam showed an increased risk of neonatal mortality among mothers living farthest away from a health facility [6]. In another study, conducted in Kenya, it was found that younger children and children with more severe illnesses travelled a further median distance to a health facility compared with older children with less serious illnesses; the rate of clinic visits decreased linearly at 0.5 km intervals and stabilised beyond 4 km [7]. These findings, unlike ours, are in keeping with the suggestion that out of pocket expenses may be a barrier to adherence, given that longer travel distances imply greater expense [23].

Some study limitations should be acknowledged. Firstly, the sample size was small. Thus, even though a trend of distance decay was seen among patients who have had MVRs, the association failed to reach statistical significance. Secondly, the relatively short median distances travelled by participants in Cape Town may limit the effect of distance decay on patient habits; it is possible that distance decay may be a factor in median distances of more than 50 km. Lastly, this study only focused on one of the many factors that influence adherence (travel distance only, but not considering out of pocket expenses, the use of public transport and patient waiting times at respective facilities to mention but a few. Given the scope of the data in the registry, information related to the mode of transport used to health facilities was not recorded. However, anecdotally most patients attending public health facilities use public transport, which incorporates an extensive network of taxi services with travel times per km being largely uniform. Further factors influencing patient INR maintenance could be gleaned by future studies incorporating qualitative methods to evaluate issues such as socio-economic status.

In summary, GIS has proven to be a useful tool for evaluating the utilization of clinics for INR monitoring. This study indicates that there is perhaps a need to establish INR facilities within existing clinics closer to patients’ residences; if patients utilize a clinic in their neighborhood or nearest to their place of residence, it could reduce travelling time and out of pocket expenses associated with transport costs and facilitate their access to healthcare. Furthermore, the load on already over-burdened clinics would be more appropriately spread.

## Conclusions

The results of this study do not provide evidence for an association between the distance from RHD patients’ residences to their INR clinics and the maintenance of INR therapeutic ranges, irrespective of whether patients had had MVRs or not. This finding was consistent among patients attending the tertiary hospital and community-based clinics. Patients in our cohort were not found to utilize the clinic closest to their residence, but instead travelling on average up to three times further for their INR monitoring.

## Abbreviations

RHD: Rheumatic heart disease
INR: International normalized ratio
REMEDY: Global Rheumatic Heart Disease Registry
GIS: Geographical information systems
GSH: Groote Schuur Hospital
MVR: Mechanical valve replacement
CHC: Community health clinic
TTR: Time within therapeutic range
